# Global research output in the health of international Arab migrants (1988–2017)

**DOI:** 10.1186/s12889-018-5690-4

**Published:** 2018-06-18

**Authors:** Waleed M. Sweileh

**Affiliations:** Department of Physiology and Pharmacology/Toxicology, College of Medicine and Health Sciences, Nablus, Palestine

**Keywords:** Bibliometric analysis, Arab, International migrants, Middle East, SciVerse Scopus

## Abstract

**Background:**

In the past few decades Arab countries had witnessed several intra-regional conflicts and civil wars that led to the creation of millions of refugees and migrants. Assessment of research activity is an indicator of national and international efforts to improve the health of those millions of war victims. Therefore, the aim of this study was to analyze published literature in international Arab migrants.

**Methods:**

Literature in international Arab migrants published during the past three decades (1988–2017) was retrieved using Scopus database. A bibliometric analysis methodology was implemented on the retrieved data. Author keywords were mapped using VOSviewer program.

**Results:**

In total, 1186 documents were retrieved. More than half (658; 55.5%) were published in the last five years (2013–2017). Retrieved documents received an average of 8.6 citations per document and an *h*-index of 45. The most frequently encountered author keywords were refugees and mental health–related terms. Three countries in the Middle East; Jordan, Lebanon, and Turkey, were among the most active countries. In total, 765 *(63.7%)* documents were about refugees, 421 (35.5%) were about migrant workers, 30 (2.5%) were about asylum seekers, and 7 (0.6%) were about trafficked and smuggled people. When data were analyzed for the nationality of migrants being investigated, 288 (24.3%) documents were about Syrians, 214 (18.0%) were about Somali, 222 (18.7%) were about Arab or Middle Eastern in general, and 147 (12.4%) were about Palestinians. The *American University of Beirut* ranked first with 45 (2.4%) publications. The most active journal in publishing research in this field was *Journal of Immigrant and Minority Health* (35; 3.0%) followed by *Journal of Refugee Studies* (23, 1.9%), *The Lancet* (19, 1.6%) and *BMC Public Health* (16, 1.3%). Publications from Jordan and Lebanon had the highest percentage of international research collaboration.

**Conclusion:**

Research in international Arab migrants showed a dramatic increase in the last few years mostly due to the Syrian war. Both mental health and Syrian refugees dominated the literature of international Arab migrants. Research in infectious diseases was relatively low. Research on non-refugee migrants such as workers, trafficked victims, and asylum seekers was also relatively low.

**Electronic supplementary material:**

The online version of this article (10.1186/s12889-018-5690-4) contains supplementary material, which is available to authorized users.

## Background

Currently, there are 22 Arab states that occupy the area stretching from the Atlantic Ocean to the Arabian Sea [1]. The total combined number of population in Arab countries is about 422 million [1]. The Arab states had witnessed several intra-regional conflicts over the past several decades. Major conflicts in the Arab region include Arab – Israeli conflict, Iraq – Iran war, Gulf war, Lebanese civil war, Palestinian intifada against Israeli occupation, Algerian civil war, Western Sahara conflict, Sudanese civil war, Yemen civil war, Somali civil war, Iraqi war, Arab spring, Syrian civil war, Libyan civil war, in addition to spread of terrorist and violent activities in the Arab region [2–7]. Conflicts, civil wars, lack of democracy, oppression, and economic instability in many Arab countries created large numbers of Arab migrants and refugees to neighboring rich Arab Gulf countries as well as to Europe, North America, and Australia [8, 9].

In this study, the term “international migrant” was used to refer toforcibly displaced people (refugees, asylum seekers, human trafficking, and smuggled people) and migrant workers [10]. The exact number of international Arab migrants is not exactly known. But there are 4.5 million Palestinian refugees living in camps inside and outside Palestine. Similarly, there are more than 5 million Syrian refugees living outside Syria. In 2015, about 41% of forcibly displaced Arab migrants were living in just four countries: Turkey, Jordan, Lebanon and Iran. Regarding international non-displaced Arab migrants, the majority were living in Saudi Arabia (10.2 million), and United Arab Emirates (8.0 million), Kuwait (2.9 million) and Oman (1.8 million) [10–12].

The health needs of migrant population are tremendous because of their suboptimal living conditions, lack of vaccination in certain cases, crowded living places, minimal health care services and insurance, rejection and stigma from certain countries, language barriers in certain cases, cultural differences, and several other factors that could negatively affect their health and survival [13–16]. Of the migrant population, children, unaccompanied minors, pregnant women, and elderly people remain the most vulnerable categories that require continuous healthcare provision [17, 18]. Another important health dimension of the migrant population is adaptation to the culture of host countries in addition to overcoming the psychological trauma of war and difficult journey to safer places [18–21]. The provision of health to migrants requires research activity that shed lights on health problems faced by migrants in their new living environment in order to develop appropriate health services that meet their health needs.

The large numbers of international Arab migrants, the deadly journey taken by some of them, and the cultural and religious differences between those migrants and that of host countries make the health of international Arab migrants both global and regional public health issue [22–24]. To better understand this issue and to contribute positively to the health of those migrants, research activity in the health of international Arab migrants need to be assessed. Such assessment will seek to identify the volume, evolution, visibility, research networks, health topics, and national and international contribution to this field. Health policy makers, in both host countries and international health organizations, will need this research analysis and assessment for future plans to alleviate health burden imposed on this category of people. The widely used and well-established methodology to measure the quantity and quality of research output in a certain scientific subject is the bibliometric analysis which is used to provide real and concrete data on research trends and priorities [25–27]. Bibliometric analysis is becoming an important, accessible, and widely accepted method to assess national and international research productivity, international collaboration, citation analysis, research trends, and scientific development in a particular field [28–32]. Bibliometric analysis is defined as the quantitative and qualitative analysis of a set of publications on a particular disease or diseases to identify the direction of research activity and thus enhances understanding of changes in that field [33].

Based on the argument stated above, the current study was carried out to assess the volume of scientific publications related to health of international Arab migrants in order to shed more light on this topic. In specific, most active countries, authors, institutions, and journals will be assessed. Furthermore, growth of publications, most cited documents, and most frequent keywords encountered in this field will be presented.

## Methods

In this study, biliometric methodology was applied using SciVerse Scopus. SciVerse Scopus is 100% inclusive of Medline and is considered one of the largest databases that included more than 23,000 indexed journals in various disciplines including health, humanities, physical science, and social sciences [34]. Therefore, SciVerse Scopus is considered very suitable for bibliometric analysis based on the advantages it has over other databases [35]. Furthermore, the search engine of SciVerse Scopus gives a wide range of search scenarios that can allow maximum retrieval of related documents. The output obtained from Scopus can be easily exported and analyzed formost active authors, institutions, countries, journals, subject areas, citations, and keyword mapping.

The keywords used in this study were grouped into three search queries. The first research query was applied to retrieve the name of any Arab country or nationality within the article title. The use of title search rather than title-abstract search was based on the idea that title search retrieves the minimum number of false positive documents. The second research query was applied to retrieve documents with words related to the definition of international migrant used in this study (international migrants, migrant workers, forcibly displaced people, refugees, asylum seekers, trafficked people, smuggled persons). The third research query was implemented to retrieve all possible documents that have health dimension in either title or abstract or keywords. The three search queries were then combined to get the final result which was limited by duration (1988–2017) and source type (journal articles only). Additional file 1 contains the search queries with the keywords used. The keywords used were developed by the author after an extensive literature review of systematic reviews in the field of migrants, refugees, asylum seekers and trafficked people published from different world regions [36–43]. The validation of the search strategy was based on review of top 200 cited articles to make sure that no or minimum number of false positive documents were present. The author manually surveyed the top 200 cited documents for false positive documents. Whenever a false positive phrase or keyword was encountered, it was added to the exclusion step. For example, there were false positive documents discussing fish migration in Arab Gulf Sea. Therefore, in the exclusion step, the word fish was added to the search strategy to exclude any document with this word. Furthermore, there was a strong and significant relationship (*P* < 0.01), as tested by interclass correlation, between the number of articles retrieved through Scopus for active authors and the number of articles retrieved for the same authors using their Scopus profile. This was suggestive of the high validity of the search strategy.

Retrieved literature was analyzed by exporting the data from Scopus to Excel software for calculation and tabulation. The exported data included the following information: number of published documents in each year, types of published documents, subject areas, names of journals, names of authors, country and institutional affiliation of authors who participated in publishing the retrieved literature. For citation analysis, Scopus provides the number of citations received for each article and allows the sorting of articles based on the number of citations. Furthermore, the Hirsh (*h*-index) of any group of retrieved documents can be provided by Scopus for comparative purposes. The *h*-index is an author-level metric that attempts to measure both the research productivity and citation impact of the publications of a scientist or a country or an institution [44]. Scopus also allows the export of all retrieved data to excel for purposes pertaining to geographical mapping or visualization of author keywords or author networking or country collaboration. Growth of publications and statistical analysis were carried out using the Statistical Package for Social Sciences (IBM SPSS statistics; version 21; Armonk, N.Y: IBM Corporation). VOSviewer software (version 1.6.8; Leiden University, the Netherlands) was used to create visualization maps, while ArcMap 10.1 (ESsri, California, USA) was used to plot the geographical distribution of the retrieved documents [45–47].

For data presentation, tables were created for top 10 active authors, countries, and institutions. The same was applied for most highly cites articles where only the top 10 cited articles were presented. For author keyword visualization, only keywords with a minimum occurrence of 10 were shown on the map. Same applies to author networking where only top 10 active authors were mapped. For international collaboration, the number of documents with authors from the same country (single country publication (SCP)) and the number of documents with authors having different country affiliations (multiple country publication (MCP)) are calculated for each country and used as an indicator of the extent of international collaboration. Countries with a high percentage of MCP are ones with highest international research collaboration. The growth rate of publications (GR) was calculated using the following equation: GR = [(Ending Value - Beginning Value) / Beginning Value] * 100. For example, if the number of retrieved documents in 2016 was 100 and that in 2015 was 50, then the GR in 2016 would be (100–50/50) * 100 = 100%. The retrieved data were also processed to segregate the number of publication on Palestinians, Syrians, Iraqi, Somali, Sudanese, and others to show the share of publications for each Arabic nationality. This study has no human subjects involved and therefore no ethical approval was needed by the Institutional Review Board of An-Najah National University.

## Results

### Growth of publications

**Fig. 1:**
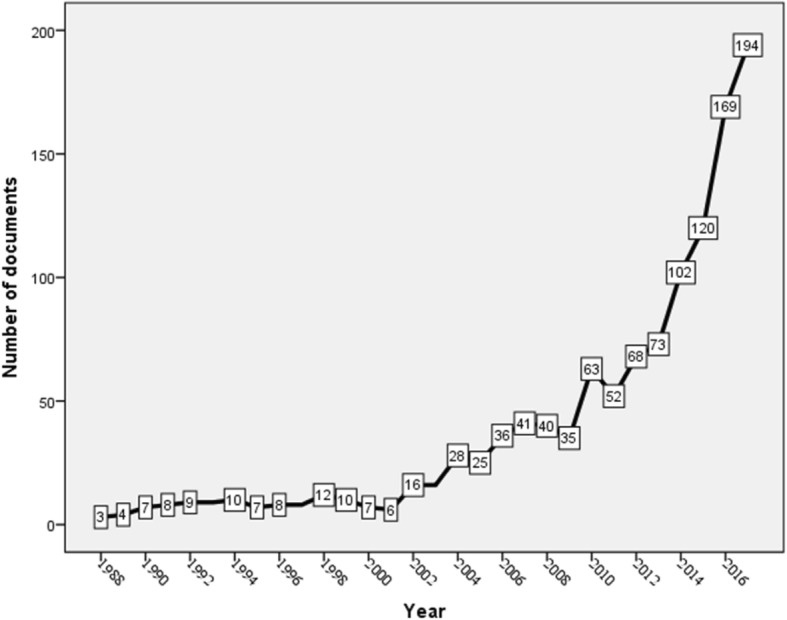
Growth of publications in international Arab migrants (1988–2017)

In total, 1186 documents discussing the health of international Arab migrants were retrieved. The highest number of documents was published in 2017 with a total of 194(16.4%) documents. More than half (658; 55.5%) of the retrieved documents were published in the last five years of the study period (2013–2017). Figure 1 shows the annual number of publications. The GR in the second decade of the study was approximately 170% while the GR in the third decade of the study was 365%.

#### Types of documents and subject areas

The majority of the retrieved documents were research articles (980; 82.6%) followed by review articles (79; 6.7%), letters (33; 2.8%), notes (25; 2.1%), editorials (17; 1.4%), conference papers (13; 1.1%), and short surveys (5; 0.4%). At the time of data analysis, there were still 34 (2.9%) documents in press and of unknown type. The language of the retrieved articles was mainly English (1115; 94.0%) followed by French (39; 3.3%), Dutch (9; 0.8%), German (8; 0.7%), Spanish (8; 0.7%) and Italian (5; 0.4%). There were four documents with Turkish/English abstracts and three with Arabic/English abstracts. The majority of documents were published in journals within the subject area of medicine (585; 49.3%), followed by those in social sciences (547, 46.1%), art and humanities (155; 13.1%), psychology (122, 10.2%), and nursing (83; 7.0%). Overlap in various subject areas created a total percentage of more than 100%.

#### Citation analysis

Retrieved documents received 10,256 citations, an average of 8.6 citations per document. The *h*-index of the retrieved documents was 45. The highest number of citations attained was 221 for a study about the psychology of resettled Sudanese refugees [48]. The most highly cited articles are shown in Table 1. The highly cited articles discussed health issues mainly for Somali and Iraqi migrants and refugees. Notably, the majority of highly cited articles were published in psychiatry, psychology, and mental health–related journals. All highly cited articles were research articles and none were review articles. Most of the highly cited articles were published during the second decade of the study period (1998–2008).

**Table 1:** Highly cited articles in health of international Arab migrants (1988–2017)

Reference	Authors	Title	Year	Source	Cited by
[48]	Schweitzer, R., Melville, F., Steel, Z., Lacherez, P.	Trauma, post-migration living difficulties, and social support as predictors of psychological adjustment in resettled Sudanese refugees	2006	*Australian and New Zealand Journal of Psychiatry*	221
[70]	Gorst-Unsworth, C., Goldenberg, E.	Psychological sequelae of torture and organised violence suffered by refugees from Iraq: Trauma-related factors compared with social factors in exile	1998	*British Journal of Psychiatry*	209
[71]	Laban, C.J., Gernaat, H.B.P.E., Komproe, I.H., Schreuders, B.A., De Jong, J.T.V.M.	Impact of a long asylum procedure on the prevalence of psychiatric disorders in Iraqi asylum seekers in The Netherlands	2004	*Journal of Nervous and Mental Disease*	151
[72]	Gerritsen, A.A.M., Bramsen, I., Devillé, W., van Willigen, L.H.M., Hovens, J.E., van der Ploeg, H.M.	Physical and mental health of Afghan, Iranian and Somali asylum seekers and refugees living in the Netherlands	2006	*Social Psychiatry and Psychiatric Epidemiology*	138
[73]	Ellis, B.H., MacDonald, H.Z., Lincoln, A.K., Cabral, H.J.	Mental Health of Somali Adolescent Refugees: The Role of Trauma, Stress, and Perceived Discrimination	2008	*Journal of Consulting and Clinical Psychology*	137
[74]	Jaranson, J.M., Butcher, J., Halcon, L., Johnson, D.R., Robertson, C., Savik, K., Spring, M., Westermeyer, J.	Somali and Oromo Refugees: Correlates of Torture and Trauma History	2004	*American Journal of Public Health*	132
[75]	Bhui, K., Abdi, A., Abdi, M., Pereira, S., Dualeh, M., Robertson, D., Sathyamoorthy, G., Ismail, H.	Traumatic events, migration characteristics and psychiatric symptoms among Somali refugees - Preliminary communication	2003	*Social Psychiatry and Psychiatric Epidemiology*	132
[76]	Laban, C.J., Gernaat, H.B.P.E., Komproe, I.H., Van Der Tweel, I., De Jong, J.T.V.M.	Postmigration living problems and common psychiatric disorders in Iraqi asylum seekers in the Netherlands	2005	*Journal of Nervous and Mental Disease*	109
[77]	McMichael, C., Manderson, L.	Somali women and well-being: Social networks and social capital among immigrant women in Australia	2004	*Human Organization*	91
[78]	Small, R., Gagnon, A., Gissler, M., Zeitlin, J., Bennis, M., Glazier, R.H., Haelterman, E., Martens, G., McDermott, S., Urquia, M., Vangen, S.	Somali women and their pregnancy outcomes postmigration: Data from six receiving countries	2008	*BJOG: An International Journal of Obstetrics and Gynaecology*	83

#### Most frequent author keywords

**Fig. 2:**
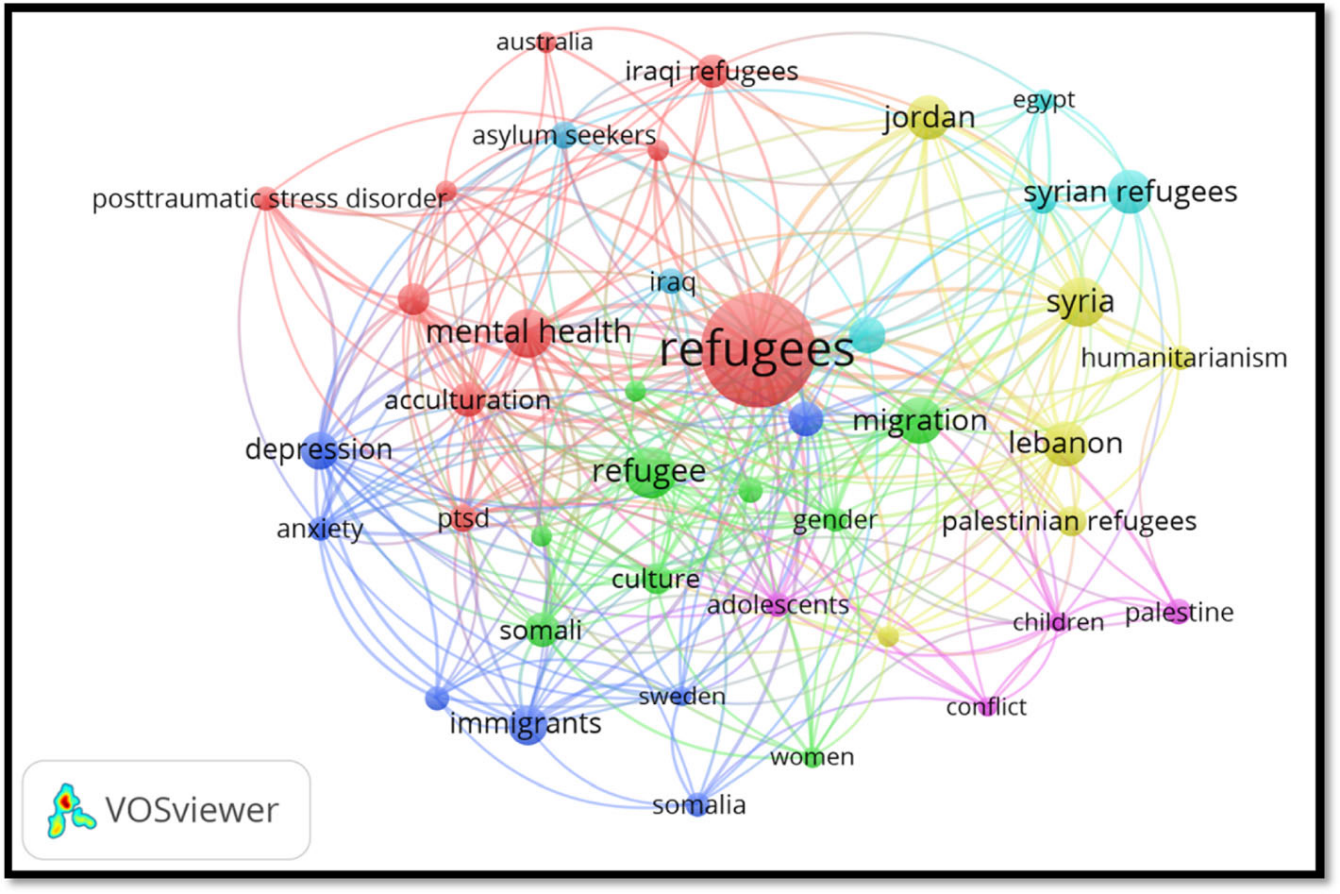
VosViewer mapping of most frequent author keywords (minimum of 10 occurrence) in international Arab migrants (1988–2017)

Analysis of occurrences of author keywords using a minimum of 10 occurrences retrieved 42 keywords distributed within seven clusters (different colors in the map). The most frequently encountered keyword was the one with the largest node size shown in the map as “refugees” (Fig. 2). The map also included (1) several mental health–related keywords; (2) names of nationalities of certain Arab migrants such as Syrian refugees, Iraqi refugees, Palestinians, Somali refugees, Sudanese, and Egyptians; (3) names of certainhost countries such as Jordan, Lebanon, and Australia; and finally (4) keywords related to asylum-seeking which has a smaller node size than that for “refugees”. In general, keywords related to migration were less common than those related to refugees.

#### Authorship analysis

In total, 2891 authors participated in publishing the retrieved documents giving a mean of 2.4 authors per article. The most active authors are listed in Table 2. Professor Jamil, H. (Wayne State University, Department of Family Medicine and Public Health Sciences, Detroit, United States) was the most prolific author in this topic. Of the most active authors, there were two authors affiliated with institutions located in Arab countries: Professor Khaled, A. affiliated with UNRWA (United Nations Relief AND Work Agency) in Jordan and Professor Khawaja, M. affiliated with the American University in Beirut. Most active authors in the field of international Arab migrants were clustered in Wayne State University, Johns Hopkins Bloomberg School of Public Health, and UNRWA health department in Jordan.

**Table 2:** Highly active researchers in health of international Arab migrants (1988–2017)

Rank^#^	Name	Number of publications (%); *N* = 1186	Affiliation
1st	Jamil, H.	18 (1.6)	Wayne State University, Department of Family Medicine and Public Health Sciences, Detroit, United States
2nd	Doocy, S.	13 (1.1)	Johns Hopkins Bloomberg School of Public Health, Baltimore, United States
3rd	Bennet, L.	11 (0.9)	Lunds Universitet, Department of Clinical Sciences, Lund, Sweden
3rd	Arnetz, B.B.	11 (0.9)	Wayne State University, Department of Family Medicine and Public Health Sciences, Detroit, United States
5th	Burnham, G.	10 (0.8)	Johns Hopkins Bloomberg School of Public Health, Department of International Health, Baltimore, United States
5th	Khader, A.	10 (0.8)	UNRWA, Department of Health, Amman, Jordan
5th	Seita, A.	10 (0.8)	UNRWA Dept. Hlth, Amman, Jordan
8th	Khawaja, M.	9 (0.8)	American University of Beirut, Beirut, Lebanon
8th	Slewa-Younan, S.	9 (0.8)	Western Sydney University, Penrith, Australia
10th	Hammad, A.	8 (0.7)	Arab Community Center for Economic and Social Services, Dearborn, United States
10th	Koponen, P.	8 (0.7)	National Institute for Health and Welfare, Helsinki, Finland
10th	Shahin, Y.	8 (0.7)	UNRWA Dept. Hlth, Amman, Jordan
10th	Aroian, K. J.	8 (0.7)	University of Central Florida, College of Nursing, Orlando, United States

^#^Authors with equal research output were given the same rank, and then a gap is left in the ranking numbers

#### Most active countries

**Fig. 3:**
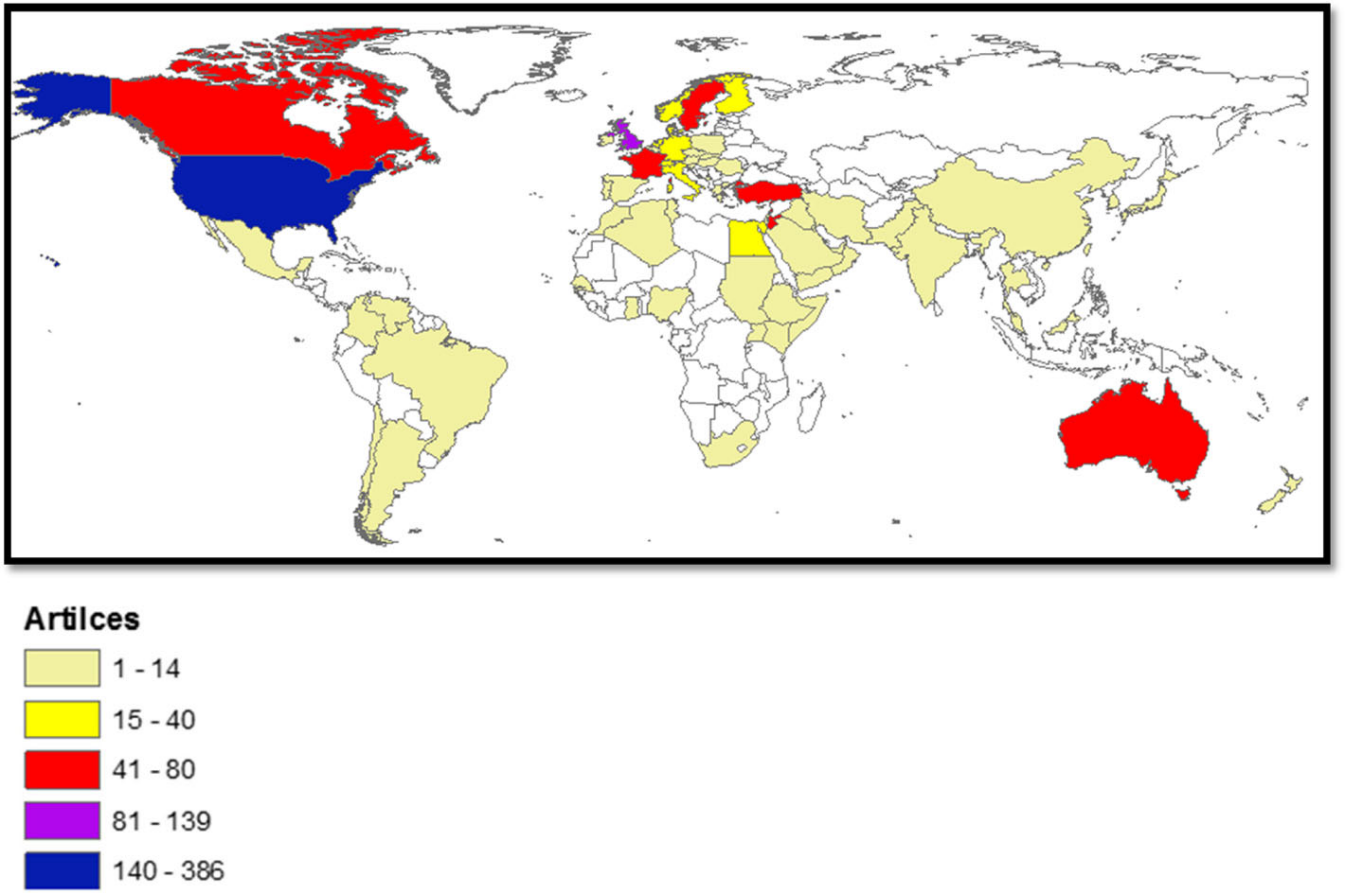
Geographical distribution of publications in international Arab migrants (1988–2017)

The USA was the most productive in this field with 386 (32.5%) documents followed by the United Kingdom (UK) (139; 11.7%). The most active list included two Arab countries; Jordan and Lebanon. The most active list also included one country in the Middle East; Turkey. Table 3 shows most active countries along with the citation per article for documents published by each country. International collaboration among the most active countries indicated that Jordan has the highest percentage (56.2%) of documents with international authors (MCP) while Australia had the lowest percentage (19.2%). The geographical distribution of publications based on the country affiliation of authors indicated that most publications about Arab migrants were coming from North America, Australia, and Western Europe (Fig. 3).

**Table 3:** Highly active countries and international research collaboration in health of international Arab migrants (1988–2017)

rank	Country	Number of publications (%) *N* = 1186	Citations per document	SCP (%)	MCP (%)
1st	United States	386 (32.6)	10.3	254 (65.8)	132 (34.2)
2nd	United Kingdom	139 (11.7)	10.3	91 (65.5)	48 (34.5)
3rd	Jordan	80 (6.5)	4.9	35 (43.8)	45 (56.3)
4th	Lebanon	73 (6.1)	9.2	35 (47.9)	38 (52.1)
5th	Australia	73 (6.0)	13.5	59 (80.8)	14 (19.2)
6th	Canada	65 (5.1)	7.9	39 (60.0)	26 (40.0)
7th	Sweden	59 (5.0)	11.1	35 (59.3)	24 (40.7)
8th	France	49 (4.1)	5.9	33 (67.3)	16 (32.7)
9th	Turkey	54 (4.1)	3.8	36 (66.7)	18 (33.3)
10th	Netherlands	40 (3.4)	18.7	24 (60.0)	16 (40.0)

^#^Equal authors have the same ranking number, and then a gap is left in the ranking numbers

SCP: single country publications

MCP: multiple country publications (international collaboration)

#### Preferred journals

The retrieved documents were published in 648 different peer-reviewed journals. The top 10 preferred journals for publishing in this field was *Journal of Immigrant and Minority Health* (35; 3.0%) followed by *Journal of Refugee Studies* (23, 1.9%), *Lancet* (19, 1.6%) and *BMC Public Health* (16, 1.3%) (Table 4). The list included seven journals in the field of migration/refugee while the remaining journals were in the field of public health and general medicine. Publications in *Journal of Refugee Studies* received the highest citations per documents (17.2 citations/document) followed by those published in *International Migration* (13.3 citations per document). Of the top 10 active journals, four had no impact factor (IF) and are not listed in the Journal Citation Report 2016 published by Thomson Reuters. Of the active journal list, *The Lancet* had the highest IF. The remaining journals had an IF less than 2.5.

**Table 4:** Most active journals in publishing articles in health of international Arab migrants (1988–2017)

Rank^#^	Journal	Number of publications (%) *N* = 1186	Citations per document	IF*
1st	*Journal Of Immigrant And Minority Health*	35 (3.0)	11.5	1.314
2nd	*Journal Of Refugee Studies*	23 (1.9)	17.2	1.143
3rd	*Lancet*	19 (1.6)	5.4	47.831
4th	*BMC Public Health*	16 (1.3)	9.4	2.265
5th	*International Migration*	16 (1.3)	13.3	0.735
6th	*Conflict And Health*	15 (1.3)	6.3	–
7th	*Journal Of Immigrant And Refugee Studies*	15 (1.3)	5.6	–
8th	*Refuge*	15 (1.3)	1.6	–
9th	*Journal Of Ethnic And Migration Studies*	13 (1.1)	6.7	1.362
10th	*Refugee Survey Quarterly*	12 (1.0)	5.8	–

^#^Journals with equal research output were given the same rank, and then a gap is left in the ranking numbers

IF=Impact Factor. The value of IF was obtained from the latest version of Journal Citation Report 2016

#### Most active institutions

The top 10active institutions/organizations are shown in Table 5. The *American University of Beirut* ranked first with 45 (2.4%) publications followed by *Wayne State University* (31; 2.6%), *Johns Hopkins Bloomberg School of Public Health* (20; 1.7%), and *The University of Jordan* (20; 1.7%) The top 10 active institutions/organizations included three in the USA, two in Finland, two in Sweden, one in the UK, and two in the Arab region.

**Table 5:** Most active institutions in research about international Arab migrants (1988–2017)

Rank^#^	Institution	Number of publications	% N = 1186	Country affiliation
1st	American University of Beirut	45	3.8	Lebanon
2nd	Wayne State University	31	2.6	USA
3rd	Johns Hopkins Bloomberg School of Public Health	20	1.7	USA
3rd	The University of Jordan	20	1.7	Jordan
5th	Uppsala Universitet	18	1.5	Sweden
5th	Lunds Universitet	18	1.5	Sweden
7th	University of Oxford	16	1.3	UK
8th	Helsingin Yliopisto	14	1.2	Finland
9th	United Nations Relief and Works Agency for Palestine Refugees in the Near East	13	1.1	–
9th	Turun yliopisto	13	1.1	Finland
9th	US Centers for Disease Control and Prevention	13	1.1	USA

^#^Institutions with equal research output were given the same rank, and then a gap is left in the ranking numbers

#### Mapping of health–related keywords

**Fig. 4:**
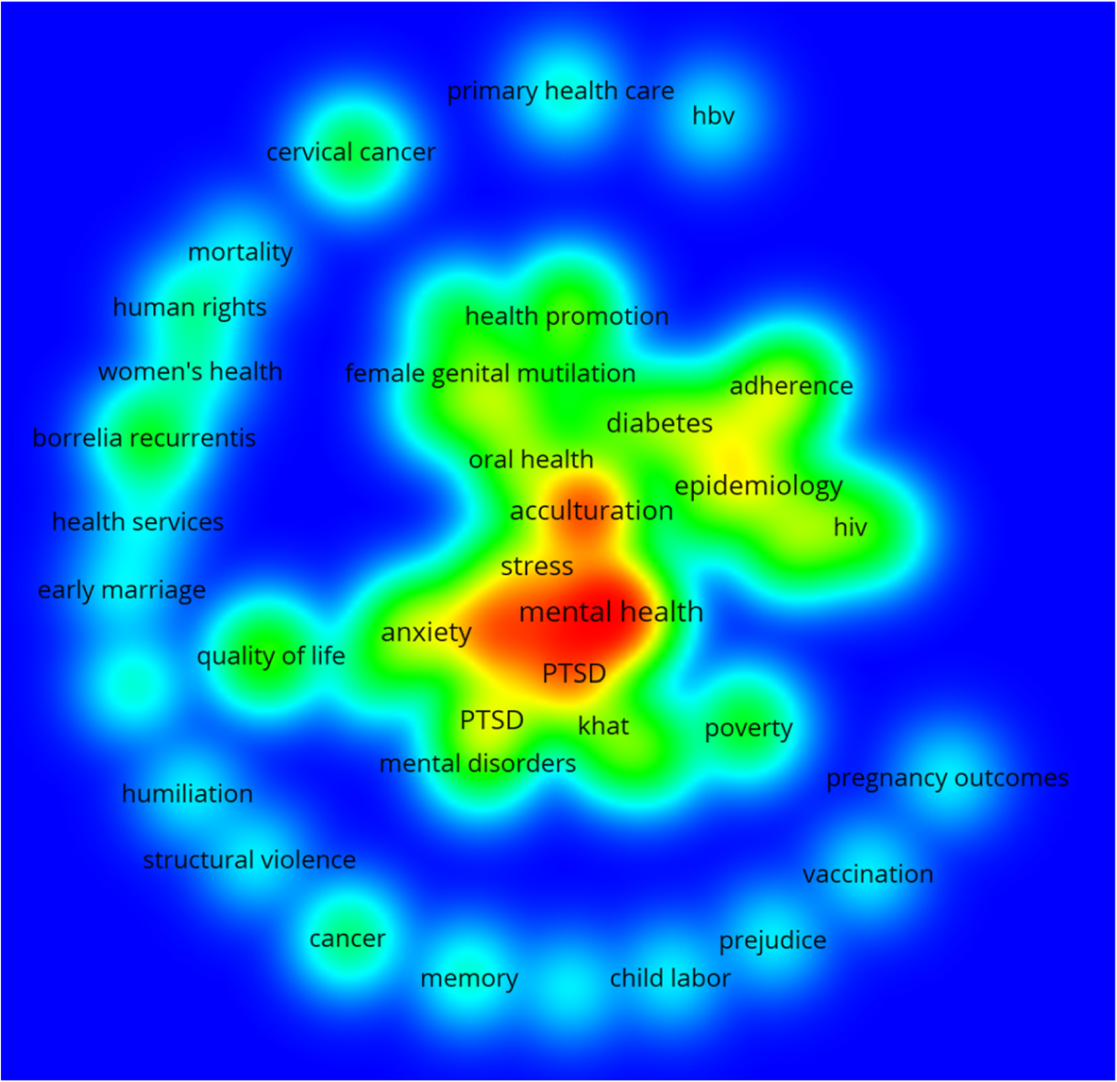
VosViewer mapping of author health – related keywords only (minimum occurrences of three) (1988–2017)

Mapping of health–related author keywords with minimum occurrences of 3 was shown in Fig. 4. The map was dominated by mental health – related keywords such as post–traumatic stress disorder (PTSD), depression, and acculturation. However other less commonly encountered keywords included ones pertaining to women’s health such as female genital mutilation (FGM), early marriage, maternal health, obstetric outcome, and cesarean. Keywords related to non-communicable diseases such as diabetes mellitus, cancer, and hypertension were also present in the map but with less dark red color indicating less frequent occurrences. Keywords related to communicable diseases such as HIV, vaccination and hepatitis were also present. Analysis of retrieved documents revealed that 450 (37.9%) articles were in the field of mental health, 255 (19.0%) were in the field of maternal and reproductive health, 124 (10.5%) were in non-communicable diseases, 35 (3.0%) in infectious diseases, and the remaining documents were in the field of health policy and systems as well as general public health.

#### Typology and nationalities of migrants

**Fig. 5:**
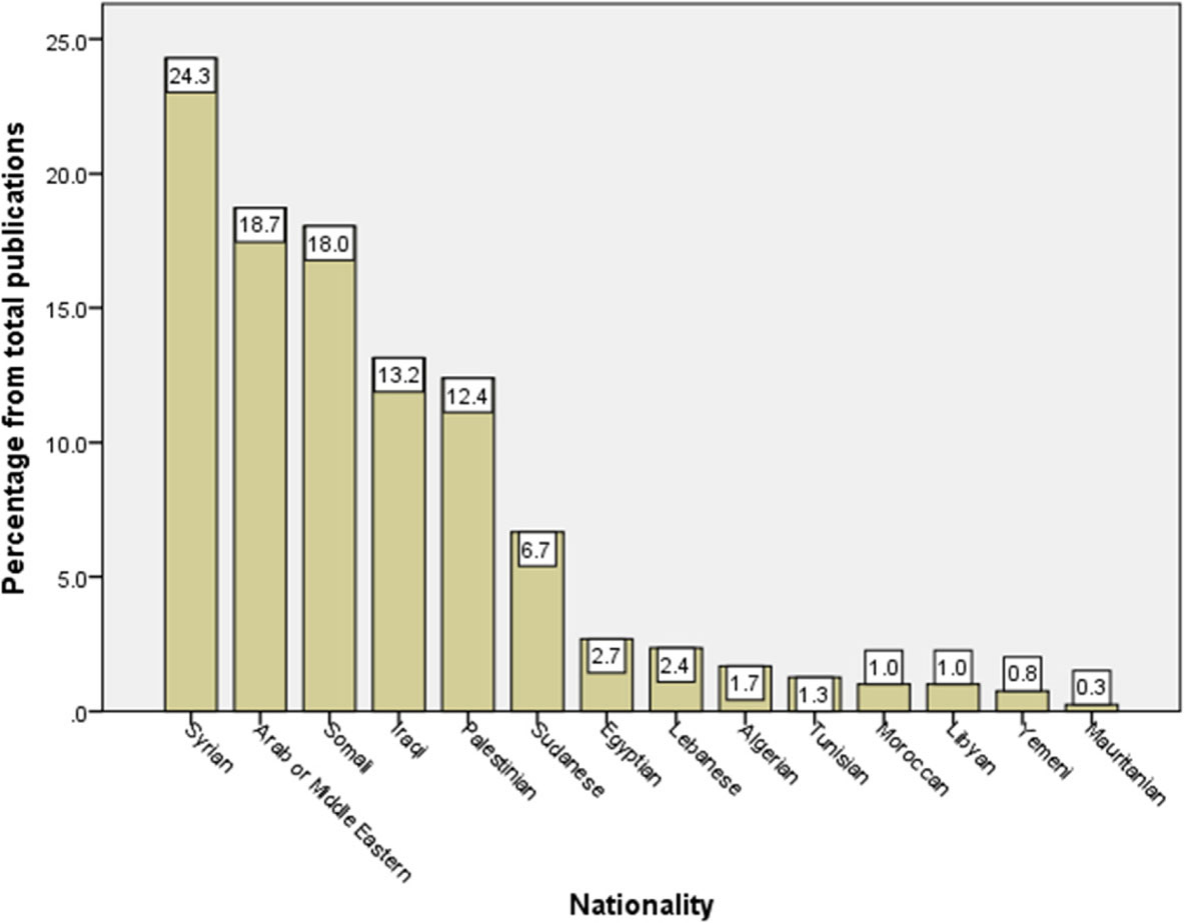
Number of publications assigned for each Arab nationality in literature pertaining to health of international Arab migrants (1988–2017)

Analysis of the retrieved documents regarding typology of Arab international migrants indicated that 765 *(63.7%)* documents were about refugees, 421 (35.5%) were about migrant workers, 30 (2.5%) were about asylum seekers, and 7 (0.6%) were about trafficked and smuggled people. When data were analyzed for nationality of migrants being investigated, 288 (24.3%) documents were about Syrians, 214 (18.0%) were about Somali, 222 (18.7%) were about Arab or Middle Eastern in general, 147 (12.4%) were about Palestinians, 156 (13.2%) were about Iraqi, 79 (6.7%) were about Sudanese, and the remaining documents were about other Arab nationalities. Figure 5 shows the frequency of retrieved documents for each Arab nationality taking into consideration that the total percentage exceeded 100% due to the fact that some articles might discuss two types of nationalities at the same time.

## Discussion

In this study, we aimed to shed light on research activity on the health of international Arab migrants. The findings of our study indicated that (1) the research activity in this field showed a dramatic increase in the last few years, most probably, due to the Syrian refugee crisis; (2) research activity related to mental health dominated the literature in international Arab migrants; (3) refugee-related research activity dominated the field on the expense of other types of international migrants, finally (4) research about the Syrians and Somalis were most frequently encountered with less emphasis on other nationalities, particularly Arabic migrants from North Africa.

The issue of Arab migration started long before the “Arab Spring”. The Palestinian refugee crisis started in 1948 after the Israeli – Arab war which led to hundreds of thousands of Palestinians leaving their homeland searching for safer places in Jordan, Lebanon, Syria, and even in remote places inside Palestine such as Gaza [49]. The Palestinian refugee problem is still existing and the Palestinian – Israeli peace process failed to find solutions for more than one million Palestinian refugees living in refugee camps in poor health conditions in Jordan, Lebanon, Syria, and other places. Our study showed that research on Palestinians ranked fourth in commonly researched Arab nationalities in the context of international migrants. This is not surprising given that the civil war in Syria dominated the political scene in the Middle East and dominated and attracted a lot of attention due to deadly journeys taken by Syrian refugees across the sea to Europe and other countries [50]. Both Palestinian and Syrian refugee crisis and the temporary allocation of those refugees in camps in Jordan and Lebanon explained our findings which showed that both Lebanon and Jordan ranked among most active countries in health–related research about international Arab migrants. Another refugee and migration issue in the Arab world was that of Somali people. The Somali civil war started in the late 1980s and is still ongoing [51]. The Somali conflict had created hundreds of thousands of refugees and migrants in neighboring African countries as well as in the USA, Europe and other places [52]. On top of all previously mentioned conflicts and wars, the Iraqi Gulf war, both the first and second one, which created large numbers of refugees, migrants and asylum seekers [53]. Unfortunately, Iraq did not yet reach a state of political stability and security to encourage the safe return of millions of Iraqi migrants.

The never-ending conflicts and wars in the Arab region had created a problem for Arab countries as well as to neighboring ones. The millions of refugees in Jordan, Lebanon, Egypt, and countries close to areas of unrest had created a public health to host countries. Without the humanitarian aids, countries with limited resources cannot afford to provide healthcare services to all categories of migrants and refugees. The spread of infectious diseases had been reported in several refugee camps. The poor sanitation, food insecurity, lack of vaccination, and over-crowdedness had created an environment for the spread of infections in refugee camps. Furthermore, poverty had driven some women to prostitution and unsafe sex with the risk of HIV/AIDS and other viral diseases. Our finding showed that research on communicable disease in international Arab migrants was dwarfed by other fields, particularly the mental health field. The bloody scene of wars and unsafe and fearful journey of migrations lefts psychological scars which affected their overall health [54, 55]. Children, women, and elderly people were most vulnerable categories to tragedies of wars and loss of beloved ones. Therefore, it was not surprising to find out a good bulk of research activity to be focused on PTSD, depression, and other mental health issues. A noticeable bulk of literature was also retrieved about maternal and reproductive health. For example, the practice of female genital mutilation among some Arab African migrants like Somalis and Sudanese was encountered [56–60]. Early marriage or forced marriage of young girls in refugee camps or among migrant families was also encountered [61, 62]. The Arabic and Islamic traditions made the adaptation of Arab migrant women in the new Westernized and open cultures uneasy which might explain some cases of early marriage among Arab migrants [63–67]. The maternal health was also encountered as one important health dimension of women, particularly in refugee camps where basic and essential maternal health care is inadequate which impose dangers on both women and newborns [68, 69].

Although refugees from Syria, Palestine, Iraq, Somali and other Arab countries represent an acute problem that requires immediate international action on all fronts, it should be emphasized that there are other millions of Arab migrants all over the world and even in the Arab Gulf. Over the past few decades, millions of Arabs have migrated from their original countries to Arab Gulf countries seeking for better and secure life. Those migrants, who are mostly workers and labor force, received one third of research efforts despite that they suffer from similar problem posed on refugees. Arab migrant workers in the Arab Gulf, USA, Europe, and Australia suffer from inequalities and sometimes being stigmatized due to the on-going war against some Islamic violent groups in the region. The cultural adaptation of those migrant workers, their lifestyle, and their health education regarding chronic diseases such as diabetes mellitus, hypertension, cancer, gynecological problems deserve more research focus and attention. Surprisingly, the contribution of Arab countries to research on international Arab migrants was inadequate. It seems that the Arab Spring in several Arab countries had disturbed the academic and research capabilities of scholars in these countries. Furthermore, it seems that Arab governments are not strengthening and supporting research about health and social challenges of Arab migrants in destination countries. The issue of migration health does not seem to be a priority in Arab countries. The Arab Gulf countries with plenty of academic and financial resources were not within the active list despite the presence of millions of Arab migrant workers in these Arab Gulf countries. The human rights and health-related challenges of these workers and their families need to be researched and incorporated into national plans to save lives and improve quality of life of migrants.

Our study is the first bibliometric study about international Arab migrants. However, our study has a few limitations. First, the use of Scopus database is a point of strength and a point of weakness because some publications on Arab migrants in unindexed Arab journals might have been missed. It should be emphasized here, that no specific journal on refugees and migration is issued from any Arab country despite that major refugee crisis in the past three decades originated from the Middle Eastern and Arab countries. Furthermore, the bulk of journals issued from Arab countries are not indexed in Scopus database. The only indexed health journal in the Middle East that publishes Arabic abstract is *Eastern Mediterranean Health Journal* which is published by WHO. This might explain the very low percentage of documents in Arabic language. A second limitation in the current study is the possibility of the presence of low percentage of false positive or false negative results which is a common limitation inherent to bibliometric methodology.

## Conclusion

International Arab migrants are distributed in many world regions and their number is increasing with time due to unstable political and military environment in Arab countries. Researchers from Arab countries and from host countries need to spend more research efforts to dig into health problems of Arab migrants. Furthermore, researchers need to focus on infectious diseases, non-communicable diseases, women’s health, and reproductive health of Arab migrants in addition to the mental health aspects of those people. Finally, migrant workers also need better attention and continuous research. The refugee crisis should not completely overshadow other categories of international migrants. Health policy makers and politicians in the Arab world need to be aware of the health needs of international Arab migrants and collaborate with destination countries to improve the health of Arab migrants. Finally, the current study endorses the Arab League in its efforts to improve the health of Arab migrants through research collaboration and networking that would ultimately raise the health standards of Arab migrants in various world regions.

## Additional file

Additional file 1: Search strategy. The file includes the keywords and search strategy implemented to retrieve the required literature (DOCX 13 kb)

## Abbreviations

IRB: Institutional Review Board
UNRWA: United Nations Relief and Works Agency
