# Supplementary material for: Global research output in the health of international Arab migrants (1988–2017)
Source: BMC Public Health. 2018 Jun 18;18:755. doi: 10.1186/s12889-018-5690-4 (PMC6006754; doi:10.1186/s12889-018-5690-4)
Supplement: Supplementary file 1 — Search strategy. The file includes the keywords and search strategy implemented to retrieve the required literature (DOCX 13 kb) [file 12889_2018_5690_MOESM1_ESM.docx]

**Additional file 1**

**Search strategy and keywords used**

**Global research output in health of international Arab migrants (1988 – 2017)**

| **strategy** | **Keywords used** | **Component** |
| --- | --- | --- |
| Title search | syria* OR leban* OR palestin* OR jordan* OR egypt* OR "middle east*" OR arab* OR morrocc* OR tunisi* OR iraq* OR kuwaiti OR "saudi* *migra*" OR yemen* OR sudanese OR libya* OR algeri* OR somal* OR mauritan* OR kuwaiti OR maghrebi | Arab nationalities |
| **AND** | | |
| Title / Abstract search | *migrant* OR *migrat* "international *migrant*" OR "international *migration" OR refugee* OR “displaced people” OR “displace person*” OR “asylum seek*” OR " displaced people" OR "stateless person" OR "exile" OR "uprooted person" OR "asylum process" OR "Asylum - seek*" ) OR TITLE ( asylum AND seek* ) OR TITLE ( "forced labour" OR "forced labor" OR "forced prostitution" OR "sexual slavery" OR "forcibly displaced" ) OR (TITLE(“migrant or *migra*) AND TITLE-ABS-KEY(work* or labor or labour or agric* or industry*) OR TITLE-ABS(“migrant work*”) | Types of international migrants investigated |
| Source title search | "International Journal of Migration, Health and Social Care" OR "Journal of Immigrant Minority Health" OR "Refugees and Human Rights" OR "Journal of Immigrant and Refugee Studies" | OR |
| **AND** | | |
|  | Limit to subject areas pertaining to health (medicine, nursing, psychology, health, pharmacology, biochemistry, microbiology) | Health component |
| **AND NOT** | | |
|  | Exclusion based on manual search |  |
| **Limit to** | | |
|  | Time period: 1988 – 2017  Source type: journal article |  |
